# Understanding the Public’s Attitudes Toward COVID-19 Vaccines in Nottinghamshire, United Kingdom: Qualitative Social Media Analysis

**DOI:** 10.2196/38404

**Published:** 2023-03-29

**Authors:** Leah Ffion Jones, Stefanie Bonfield, Jade Farrell, Dale Weston

**Affiliations:** 1 UK Health Security Agency Salisbury United Kingdom; 2 Nottingham City Council Nottingham United Kingdom

**Keywords:** COVID-19, vaccine, social media, qualitative, vaccine hesitancy, infodemic, misinformation, infodemiology, online health information, content analysis, Facebook, Twitter, transmission

## Abstract

**Background:**

COVID-19 vaccines remain central to the UK government’s plan for tackling the COVID-19 pandemic. Average uptake of 3 doses in the United Kingdom stood at 66.7% as of March 2022; however, this rate varies across localities. Understanding the views of groups who have low vaccine uptake is crucial to guide efforts to improve vaccine uptake.

**Objective:**

This study aims to understand the public’s attitudes toward COVID-19 vaccines in Nottinghamshire, United Kingdom.

**Methods:**

A qualitative thematic analysis of social media posts from Nottinghamshire-based profiles and data sources was conducted. A manual search strategy was used to search the Nottingham Post website and local Facebook and Twitter accounts from September 2021 to October 2021. Only comments in the public domain and in English were included in the analysis.

**Results:**

A total of 3508 comments from 1238 users on COVID-19 vaccine posts by 10 different local organizations were analyzed, and 6 overarching themes were identified: trust in the vaccines, often characterized by a lack of trust in vaccine information, information sources including the media, and the government; beliefs about safety including doubts about the speed of development and approval process, the severity of side effects, and belief that the ingredients are harmful; belief that the vaccines are not effective as people can still become infected and spread the virus and that the vaccines may increase transmission through shedding; belief that the vaccines are not necessary due to low perceived risk of death and severe outcomes and use of other protective measures such as natural immunity, ventilation, testing, face coverings, and self-isolation; individual rights and freedoms to be able to choose to be vaccinated or not without judgement or discrimination; and barriers to physical access.

**Conclusions:**

The findings revealed a wide range of beliefs and attitudes toward COVID-19 vaccination. Implications for the vaccine program in Nottinghamshire include communication strategies delivered by trusted sources to address the gaps in knowledge identified while acknowledging some negatives such as side effects alongside emphasizing the benefits. These strategies should avoid perpetuating myths and avoid using scare tactics when addressing risk perceptions. Accessibility should also be considered with a review of current vaccination site locations, opening hours, and transport links. Additional research may benefit from using qualitative interviews or focus groups to further probe on the themes identified and explore the acceptability of the recommended interventions.

## Introduction

### Background

COVID-19 vaccines offer substantial protection against severe illness, hospitalization, and mortality [1] and remain central to the UK government’s plan for tackling the COVID-19 pandemic [2]. Although the vaccine rollout has been widespread, its effectiveness in reducing negative public health outcomes relies on high levels of equitable uptake across the general population [3]. It is therefore important to identify specific groups of individuals for which uptake is low to guide targeted efforts toward understanding how uptake can be improved. Low vaccine uptake has been linked to deprivation across all groups and ethnicities, and common reasons for hesitancy include low risk perception, concerns about the speed of vaccine development, side effects, belief in conspiracy theories, and low levels of trust in the government [4,5].

As of March 8, 2022, the average uptake of 3 doses in the United Kingdom was 66.7%. However, there are regions and localities in the United Kingdom where uptake falls considerably lower than the UK average [6]. One particular region with an average below the national is Nottingham, where only 43.2% have received 3 doses of the vaccine [7]. The wider county is made up of 7 boroughs with a total population of around 824,800 people, 21% of whom are over 65 years old and just 4% belong to Black and minority ethnicities (compared with the 15% national average) [8]. Preliminary local insights also suggest there is a large proportion of vaccine-hesitant individuals, as well as strong levels of antivaccine sentiment and low trust in the National Health Service (NHS) among less affluent communities across Nottinghamshire [9].

Although a range of interventions to increase vaccine uptake has been implemented across Nottingham city in a range of formats and languages, vaccine uptake remains relatively low. In collaboration with Nottingham City Council, we were eager to understand why vaccine hesitancy persists in the Nottinghamshire region. Importantly, previous research has found that antivaccine information in social media posts can increase vaccine hesitancy [10]. Thus, it is important to understand the nature of views about the vaccine that are visible to people living in Nottinghamshire on social media and to explore the drivers of vaccine hesitancy in this population. This paper details a rapid thematic analysis of comments from the general public on online posts about COVID-19 vaccines in Nottinghamshire.

### Aims

The aim of this work was to use social media data to understand the attitudes and behaviors of Nottinghamshire residents toward COVID-19 vaccination, including any barriers and facilitators to uptake. The findings will be used to make recommendations for increasing uptake and reducing vaccine hesitancy in Nottinghamshire.

## Methods

### Design

We performed a qualitative thematic analysis of social media posts from Nottinghamshire-based profiles and data sources. A pragmatist approach was used to rapidly analyze user comments following the framework method [11].

### Data Sources

Publicly available qualitative comments from members of the public in local online posts about COVID-19 vaccines were collected from 3 online sources: a local online newspaper called The Nottingham Post and local accounts on 2 social media platforms (Facebook and Twitter).

The Nottingham Post website (Nottinghamshire Live) posts newspaper articles on which registered readers can comment. Comments are publicly available to all readers regardless of registration status.

Facebook and Twitter are social media platforms that allow users to post short messages (posts and tweets, respectively) on which other users can comment. After consulting with our Nottingham City Council collaborators, the research team agreed that posts and tweets from 13 different local organizations (including Nottingham Post) would be searched for vaccine-relevant content (see Textbox 1).

**Local organizations included in the search criteria.**

1. Nottingham Post
2. BBC Radio Nottingham
3. Nottingham and Nottinghamshire Clinical Commissioning Group (CCG)
4. Nottingham County Council
5. Nottingham City Council
6. Ashfield Council
7. Broxtowe Council
8. Bassetlaw Council
9. Mansfield Council
10. Newark and Sherwood Council
11. Nottinghamshire Healthcare National Health Service (NHS) Foundation Trust
12. Gedling District Council
13. Rushcliffe Borough Council

### Ethical Considerations

The UK Health Security Agency research ethics and governance group was consulted about this piece of work and concluded that ethical approval would not be required as the data are entirely within the public domain. The committee advised the research team to ensure that all comments be anonymized and not contain any identifiable data.

### Search Criteria and Comment Inclusion Criteria

In October 2021, a manual search strategy was used to identify vaccine-relevant posts. The following search strategy was developed: article title, Facebook post, or Tweet containing one or more of the following phrases: ‘vaccin*’ OR ‘vax’ OR ‘jab’ OR ‘Pfizer’ OR ‘Astra Zeneca’ OR ‘AZ’ OR ‘Moderna’ OR ‘passport’ OR ‘Covid Pass’ within the following date range: September 7, 2021, to October 6, 2021.

To scope the amount of data available, 1 researcher (SB) used this initial search criterion to manually identify the number of articles and associated comments on Nottingham Post articles (website only). This identified 4370 comments in total, which the research team deemed plausible for reaching thematic saturation and a manageable number of comments to feasibly analyze. Note, at this stage, comments were not subject to any criteria and were not examined nor analyzed.

Comments were included in the analysis if they met the following criteria. First, comments were available in the public domain. Comments on the Nottingham Post are visible to all readers, regardless of whether they have registered for an account, and we included all Facebook and Twitter posts that were open and public that could be accessed and commented on by anyone with a Facebook or Twitter account. Second, comments included text written in English.

Comments containing only emojis or the names of other individuals, by way of tagging another user, were excluded from the analysis.

### Data Collection

All comments meeting the inclusion and exclusion criteria were manually copied and pasted from the respective online platforms into an Excel spreadsheet and categorized by the following details: organization (eg, Nottingham Post); original article, post, or tweet title; and date, username, user comment, and user comment date.

Once all comments were collected and formatted within Excel, names or usernames were replaced with anonymized user numbers, and names or usernames within comments were depersonalized by using their corresponding user number or removing completely; therefore, it would not be possible to identify any individual users from the data analyzed. The anonymized Excel file was imported into NVivo (version 2020) for analysis.

### Analyses

Two members of the research team (SB and LFJ) analyzed the data. LFJ is a female behavioral scientist with a doctorate in health psychology and substantial experience leading qualitative research in several areas of public health. SB is a female behavioral science research assistant with experience supporting qualitative research projects. At the time of the study, both researchers were working on various projects related to testing uptake and vaccine hesitancy during the COVID-19 pandemic.

A 6-step thematic framework approach was used to rapidly analyze the data as described in the following paragraphs [12,13]. The research team regularly discussed the nature of the social media comments during the copying and pasting stage of data collection (Step 1). SB and LFJ independently and inductively coded 10% of the data (5% of the comments each). The researchers then met to discuss and refine their respective coding frameworks and agree on a coding criterion. This process was repeated after 40% and 70% of the data had been coded and each researcher had analyzed 50% of the data each using their coding framework (Step 2). Any data unrelated to the research question were discarded and not analyzed (eg, insults, advertising, tributes of respect, non-COVID-19 vaccine–related topics).

After 70% of the data had been coded, both researchers agreed that they were approaching saturation [14] and developed potential themes from the codes they had generated (Step 3). They then proceeded to only code novel information from the remaining 30% of data. Once coding was complete, the researchers refined their respective coding frameworks and themes (Step 4). The themes and codes were then combined in NVivo, and the researchers met to discuss and agree on a final structure for organizing and labelling the data (Step 5). One researcher produced an initial narrative of the study results, and this was checked for accuracy and finalized by the second researcher (Step 6).

Regular communication between the 2 researchers who analyzed the data was maintained throughout the analyses to discuss and compare approaches to developing the coding framework and the criteria used for inclusion or exclusion of a comment or code. This allowed consistency to be assessed at each stage, and variations between researchers’ approaches were addressed before progressing to the next stage of analysis.

## Results

### Descriptive Findings

A total of 3508 comments from 1238 social media users on COVID-19 vaccine posts by 10 different local organizations were analyzed. Comments were taken from 25 Nottingham Post website articles, 57 Facebook posts, and 27 tweets; see Table 1 for a summary of totals. Organizations found in Textbox 1 that are not listed in Table 1 were searched, but no relevant posts nor articles were found that met the final inclusion criteria.

**Table 1:** Comments included for analysis from each local organization and online platform.

Local organizations	Nottingham Post website			Facebook			Twitter	
	Articles (n=25), n	Comments (n=562), n	Posts (n=57), n		Comments (n=2906), n	Tweets (n=27), n		Comments (n=40), n
Nottingham Post	25	562	14		2465	9		12
My Nottingham	N/A^a^	N/A	11		36	7		9
NHS^b^ Nottingham and Nottinghamshire CCG^c^	N/A	N/A	3		5	3		4
Nottinghamshire County Council	N/A	N/A	7		26	5		10
Ashfield Council	N/A	N/A	2		52	0		0
Broxtowe District Council	N/A	N/A	1		1	2		2
Mansfield District Council	N/A	N/A	8		162	0		0
Bassetlaw Council	N/A	N/A	4		76	0		0
Newark and Sherwood Council	N/A	N/A	4		32	1		3
BBC Radio Nottingham	N/A	N/A	3		51	0		0

^a^N/A: not applicable.

^b^NHS: National Health Service.

^c^CCG: Clinical Commissioning Group.

### Framework Analysis

In order to conceal the identity of the users in this study, we refrained from using quotes to illustrate themes. Instead, we paraphrased comments where necessary and have used similar language to the users when describing results.

#### Themes

Analysis identified 6 overarching themes: trust in the vaccines; beliefs about safety; beliefs about effectiveness; belief that the vaccines are not necessary; individual choice, rights, and freedoms; and barriers to physical access. The findings presented here intend to provide an in-depth description of the negative or hesitant attitudes expressed by users toward the vaccines, which represent the vast majority of the views expressed overall.

#### Trust in the Vaccines

Although some views were favorable, many participants expressed a lack of trust in the vaccines. Negative views stemmed from a lack of trust in vaccine information, the sources of information, the government, and the government’s decisions for the vaccines and the vaccine agenda.

##### Lack of Trust in Vaccine Information and Information Sources

Many hesitant individuals believed that information about the vaccines was inaccurate or false, commenting that there was too much misinformation available and that the truth was being suppressed. For example, some thought that vaccine deaths and side effects were under-reported, believing that real vaccine death rates were higher and were being concealed. Additionally, some held the belief that COVID-19–related deaths are classified and reported in ways that inflate the real figures.

There were varied opinions on which sources of information could be trusted, although many reported a lack of trust in the media. Some believed that certain media channels were too censored and positively biased regarding information about the vaccines.

Several users suggested that media stories were used as propaganda to scare people and designed to cause greater division. For example, some believed that the media only publish articles that align with provaccine attitudes and thought that articles that report deaths of unvaccinated individuals purposefully intended to cause fear among the unvaccinated. Some also suggested that articles about the vaccines were clickbait designed to generate financial gains and did not provide sufficient details to prevent skepticism.

Some thought that only known medical professionals and scientists could be trusted to provide vaccine information. However, a few users doubted the integrity of doctors and scientists as they believed that their opinions had been suppressed or were biased by pharmaceutical interests and financial objectives.

##### Lack of Trust in Vaccine Governance

Several reported that they did not trust the government, and many commented that the government narrative had changed surrounding vaccination. For example, some users expressed that the vaccines were originally advertised as being able to prevent infection after 2 doses but now a booster is required and that this only reduces the risk of infection, rather than preventing it.

Users also expressed concerns and lack of trust in the government’s decisions, particularly around guidance and legislation.

##### Lack of Trust in the Vaccine Program Agenda

Some believed that the vaccines were rolled out to meet objectives other than improving public health, such as to reduce the population and control society. There was also a lack of trust in vaccine companies, perceiving them to be profiting from the vaccines and pursuing commercial objectives. For example, one user believed that booster vaccines had been implemented as a result of surplus doses from the initial program.

A few believed that vaccinating children was to prevent the disruption to education caused by COVID-19, rather than to protect them from the virus.

Some believed that COVID-19 or vaccination was a conspiracy, labelling it a “plandemic” or “convid.” Theories included believing that the vaccines track people’s location, the vaccines are linked to 5G, COVID-19 came from a laboratory, and COVID-19 was downloaded from a computer.

#### Beliefs About Safety

Some participants commented that the vaccines are safe. However, many believed that the vaccines were not without risks and raised concerns about the safety of their development, the impact on health, and the vaccine ingredients.

##### Vaccine Research and Development Have Been Insufficient

Several expressed concerns that the vaccines had not been sufficiently tested, with many suggesting that those currently vaccinated are unknowingly participating in an experimental trial like “guinea pigs” or “lab mice.” Some believed that the vaccines were not safe for everyone and that children should not be given the vaccines until more research had been conducted.

A common belief was that the vaccines would remain under trial until 2023 and some indicated that they would wait until the trial on the general public is over. A few expressed stronger antivaccine sentiment, labelling the vaccines as a form of experimental gene therapy and believing that they can change DNA.

It was common for users to compare COVID-19 vaccines to other vaccines and medical treatments. Some suggested that the COVID-19 vaccines had not been around as long as other medications and vaccines, had not been tested as thoroughly, and were a different type of vaccine in comparison to others.

##### The Vaccines Have Severe Health Consequences

Many suggested that the vaccines were linked to heart problems including myocarditis, heart attacks, and strokes, and some were particularly concerned about these affecting young people. There were concerns that the vaccines were causing blood clots, with some branding it a “clotshot” and others mentioning a condition that causes blood clots (vaccine-induced immune thrombosis and thrombocytopenia).

Several other severe side effects from the vaccines were mentioned including blindness, antibody dependent enhancement, fatigue, cancer, organ failure, skin conditions, kidney problems, joint and muscle pains, and autoimmunity disease, as well as concerns that the vaccines may affect women’s menstrual cycles and fertility. A few mentioned learning about side effects from reading the yellow card reporting website.

Many suggested that the vaccines cause deaths. Some believed that thousands of people had died from the vaccines and that there would be more deaths to come, with a few who believed that the vaccine program is “genocide.”

##### The Vaccines Contain Harmful Ingredients

Some believed that the vaccines contain harmful ingredients such as “poison,” “chemicals,” and “toxins.” A few were concerned about vaccines that contained mRNA.

#### Beliefs About Effectiveness

Some participants believed that the vaccines are safe and effective. However, several commented that the vaccines do not remove the risk of COVID-19, that is, that vaccinated individuals can still become infected and are still able to spread the virus to others. Some speculated that more people in hospital were vaccinated than unvaccinated. There were some who believed that the vaccines may increase transmission, suggesting that the COVID-19 vaccines cause shedding and allow the virus to mutate, giving rise to other variants of the virus.

Some were suspicious about the effectiveness and resilience of the vaccines given the requirement of a booster dose.

#### Belief That the Vaccines Are Not Necessary

Some suggested that the vaccines were not necessary, either because they did not perceive themselves to be at risk of suffering from COVID-19 or because they believed alternative options would be more effective than vaccination.

##### Low Perceived Risk of COVID-19

Some believed that severe consequences would be an unlikely result of COVID-19 for most people. For example, some thought that their chances of hospitalization or death were extremely low. These users believed that only high-risk individuals should get vaccinated and that young people and children were at particularly low risk of suffering from COVID-19. Others perceived no risk because they did not believe that COVID-19 existed at all, believing that the pandemic is not real.

Some drew comparisons between the risks of COVID-19 and risks of the vaccines. One felt that vaccine risks were greater, while others thought they were similar, noting that people can die from both COVID-19 and the vaccines and that reactions to both will be similar.

##### Alternative Solutions Are Superior to the Vaccines

Some believed that natural immunity offers stronger protection and that their immune system would keep them safe. Others thought that measures such as increasing ventilation and regular testing would be more successful in controlling the spread. Some noted the importance of continuing to engage in protective behaviors besides the vaccines such as mask wearing and self-isolation when symptomatic. A few also mentioned other potential ways to deal with COVID-19, including taking vitamin D and hospital treatment.

#### Individual Choice, Rights, and Freedoms

The findings revealed that the topic of vaccination was a controversial topic and that many are opposed to mandating the vaccine and the use of incentives.

##### Perceptions of Vaccinated and Unvaccinated Individuals

Some thought that vaccinated individuals are “sheep” following social norms, have been brainwashed, and are unknowingly part of vaccine research to test the long-term effects. A few believed vaccinated individuals should not be proud of their vaccination status and commented that people who have pressured or forced people into getting vaccinated would regret it.

There were also negative attitudes toward unvaccinated individuals, with some believing those who are unvaccinated are selfish and put others at risk. Some felt that getting vaccinated is the right thing to do and suggested that unvaccinated individuals ignore scientific advice. A few believed that the unvaccinated should not be treated by the NHS or they should have to pay if they require medical help as a result of COVID-19.

Some unvaccinated individuals voiced frustration with those expressing negative attitudes toward their decision, and some felt that they were not “anti-vax” but had legitimate concerns about the COVID-19 vaccines. Some believed that those who had concerns about the vaccines were not listened to and that unvaccinated individuals would be wrongly perceived as uneducated by those who are vaccinated.

Addressing the controversy of vaccination, some believed that people should not be concerned about other people’s vaccination status because the vaccines only offer personal protection. Others called for people not to argue or judge people based on vaccination decisions.

##### Individual Choice Versus Enforcement

Several users commented that everyone should have a right to decide whether to get vaccinated. For vaccinating children, some felt that this should be the child’s decision, and others felt that it was for the parents to decide.

Some believed that enforcing vaccines was against human rights, unlawful, and in breach of employment contracts among health care workers. Some cautioned that mandating the vaccine among care workers would lead to staff shortages, which could lead to increased pressure on the NHS, care home closures, and further lockdowns, as well as relatives needing to care for vulnerable loved ones.

##### Beliefs About Incentivizing the Vaccines

Some reported that they were incentivized to be vaccinated by being able to see friends and family and to end the pandemic.

There were concerned comments that the government was pressuring people to get vaccinated through bribes and coercion including the use of vaccine passports. Negative attitudes toward vaccine passports that were detailed in the analysis included beliefs that the government is forcing people to get vaccinated, that the government is controlling people, that passports are intended to fund wealthy companies, and that passports do not make sense when vaccinated individuals can still get infected and transmit the virus to others.

#### Barriers to Physical Access

A few commented that travelling to a vaccine center may be difficult for some and suggested that more vaccine sites should be made available and financial support be given to enable people to travel to sites.

## Discussion

### Principal Findings

For the first time, we present a qualitative study of attitudes toward the COVID-19 vaccines visible in the public domain on local Nottinghamshire organizations’ social media pages. The findings reveal a wide range of beliefs and attitudes toward COVID-19 vaccination as well as several key barriers and facilitators to getting vaccinated. The spectrum of beliefs captured reflects the notion that people are not necessarily “anti-vax” but have perceived legitimate concerns or misunderstandings about the COVID-19 vaccines, some of which could be addressed by targeted and nuanced communications.

There were perceptions that vaccine information, particularly from national and local media sources, was designed to scare unvaccinated individuals into getting the vaccine, and several suggested that they did not believe or chose to ignore information as a result. This resonates with the wider literature on the impact of fear appeals. According to the Extended Parallel Process Model [15], in situations where individuals perceive there to be limited solutions for dealing with a threat, they may resort to defensive fear control processes. An individual may deny the existence of the risk, reject the message, or even increase engagement in behaviors that contribute to the risk. Given this, it is critical that the risks are not artificially inflated or overstated and for information about the legitimate and accurate risks of a health threat (such as COVID-19) to be accompanied by clear information about the recommended behavior, emphasizing explicitly what individuals should do, why the behavior is important or effective at dealing with the threat, and how to engage in the behavior [15].

### Implications for Vaccine Programs

A number of recommendations centered around communication strategies and accessibility is presented below. Although designed on the basis of data gathered from Nottingham-relevant social media, these could also be adapted for other areas with similar population beliefs and attitudes.

Communication strategies delivered by known trusted sources are needed to tackle vaccine-hesitant beliefs. The information provided should always highlight the benefits of the vaccines but should allow for some acknowledgement of the uncertainties and the possibility of negative consequences such as side effects. Furthermore, strategies should reframe from tackling antivax views to approach strategies as tackling misunderstanding and lack of knowledge of the benefits of vaccination, as many with hesitant beliefs and vaccine concerns do not consider themselves to be “anti-vax” but consider themselves to have legitimate concerns [16].

Any communication strategy should ensure that information does not perpetuate or promote misinformation or myths. This could involve local authorities working with local media outlets to support information delivery to ensure news articles provide accurate scientific information with links for further details and monitoring of local misinformation on social media in order to inform correct information targeting. It may be necessary to ensure that local news articles provide sufficient information on a given story to prevent speculation, debates, and division among readers and commenters.

Local communication strategies should rely on local insights and any gaps in knowledge that have been identified, for example vaccine effectiveness, safety, and risk perceptions. Addressing risk perception should not rely on scare tactics, as this can backfire and cause people to act in other ways than the desired behavior or ignore the messaging [15]. By including accurate information about COVID-19 alongside vaccination information and provision of evidence that the vaccine is the best defense against COVID-19, communications can address lack of knowledge and understanding about the virus and could help foster realistic risk perceptions.

A common belief across users in this study was the belief that the decision to vaccinate is a personal choice. Acknowledging that the decision to vaccinate is a personal choice alongside emphasizing the aforementioned benefits could increase engagement.

Ensuring accessibility to all is an important factor in ensuring good vaccine uptake, including access to vaccination centers and booking systems. In the case of Nottingham, it may be beneficial to review current vaccine center locations in relation to public transport links and consider moving or opening centers into different or new localities to ensure a wide spread of accessibility across the region.

### Implications for Further Research

It is recommended that this work is replicated in other localities to gather insights and to understand localized differences. Where insights are similar, the recommendations outlined here could be used to shape and modify existing vaccination programs, and where insights differ, the recommendations will need adapting for the target population.

It would be beneficial to corroborate the insights from this work with further qualitative interviews or focus groups with the local population to probe further on some of the findings, particularly the negative beliefs about safety and effectiveness. However, engaging this population in participatory research would likely have its limitations and would not necessarily guarantee the collection of such honest views.

### Strengths and Limitations

This study involved a relatively small sample size (1238 users’ comments) that is unlikely to be representative of vaccine attitudes among Nottinghamshire’s population. However, the unfiltered and sometimes anonymous nature of social media platforms means that the data and insights present valuable insights into the broad range of views visible in the public domain to local individuals with access to social media.

The authors acknowledge that some comments may have been removed by the media platforms or the owners of the post prior to data collection. According to Nottingham Post’s house rules [17], users are asked to refrain from posting personal attacks, swearing, mindless abuse, or hate speech to avoid comment deletion. Facebook does not remove comments containing false news but does take steps to reduce its spread by showing it lower down on the newsfeed [18], and Twitter removes sensitive adult content, graphic violence, hateful imagery, violent sexual conduct, and gratuitous gore [19]. Therefore, it is likely that a wide range of views were captured during data collection including negative attitudes and misinformation. However, comments containing beliefs and attitudes about the vaccines may have been removed if they also contained content that was in breach of the platforms’ terms and conditions.

The authors also acknowledge that no demographic information relating to the social media users was available for inclusion in this study; therefore, we cannot draw conclusions that may be specific to gender, age groups, ethnicity, or Nottingham locality. It is also possible that users who commented did not necessarily live in Nottinghamshire; however, it is likely that users followed the organizations’ Facebook and Twitter pages because they live locally. Therefore, the majority of findings are likely to represent the opinions of those living in Nottinghamshire. Additionally, the findings provide valuable insight into comments about the vaccines that are publicly visible to those who follow posts from organizations in Nottinghamshire that may be influential to public attitudes more generally.

Despite the limitations associated with the data, it is important to acknowledge that, as the comments were given outside of a research setting and given the richness of the data, it is likely that the opinions and attitudes expressed are honest and unfiltered. This methodology has allowed us to access the views of individuals who may not have otherwise engaged with research or, if they had, may not have provided such honest views.

### Conclusions

This social media analysis has highlighted a number of barriers and facilitators associated with vaccine uptake in the Nottinghamshire region. The resulting recommendations should be considered where appropriate and where similar insights are found in other localities. Additional research may benefit from using qualitative interviews or focus groups to further probe on the themes identified and explore the acceptability of the recommended interventions.

## Abbreviations

NHS: National Health Service
